# A Novel PLCζ Mutation Linked to Male Factor Infertility Induces a Gain-of-Function Effect on Ca^2+^ Oscillations in Eggs

**DOI:** 10.3390/ijms26136241

**Published:** 2025-06-28

**Authors:** Alaaeldin Saleh, Zizhen Huang, Maryam Al Shaikh, Tomasz P. Jurkowski, Zeyaul Islam, Karl Swann, Michail Nomikos

**Affiliations:** 1 Department of Basic Medical Sciences, College of Medicine, QU Health, Qatar University, Doha P.O. Box 123, Qatar; 2 School of Biosciences, Cardiff University, Cardiff CF10 3AX, UKjurkowskit@cardiff.ac.uk (T.P.J.);; 3 Diabetes Research Center, Qatar Biomedical Research Institute (QBRI), Doha P.O. Box 34110, Qatar

**Keywords:** phospholipase C zeta, calcium oscillations, fertilization, ICSI, male infertility

## Abstract

Mammalian fertilization is triggered by a series of calcium (Ca^2+^) oscillations that are essential for egg activation and successful embryo development. It is widely accepted that Phospholipase C zeta (PLCζ) is the sperm-derived factor that triggers these oscillations, initiating egg activation through the hydrolysis of phosphatidylinositol 4,5-bisphosphate (PIP_2_) into inositol 1,4,5-trisphosphate (IP_3_) and diacylglycerol (DAG), leading to Ca^2+^ release. Several studies have reported a number of PLCζ mutations associated with polyspermy, egg activation failure and early embryonic arrest. Herein, six infertility-linked PLCζ mutations (I120M, L246F, L277P, S350P, A384V and M578T) spanning different domains of PLCζ were selected for characterization through in vivo assessment of their Ca^2+^-oscillation-inducing activities and complementary in silico analysis. Our data revealed that five of the investigated PLCζ mutants exhibited reduced or complete loss of in vivo Ca^2+^-oscillation-inducing activity, with the exception of the L277P, which resulted in increased frequency and duration of Ca^2+^ oscillations. Molecular modeling of PLCζ mutants was consistent with the in vivo characterization, revealing that most mutations have a deleterious effect on the structural stability. For the first time, we provide evidence that a gain-of-function PLCζ mutation may be a cause of fertilization failure in humans. Our findings suggest that PLCζ enzymatic activity must operate within an optimal range to ensure successful egg activation and early embryonic development. Additionally, we demonstrate the essential role of all PLCζ domains in maintaining the Ca^2+^ oscillation-inducing activity in eggs and the importance of PLCζ functionality in human fertilization.

## 1. Introduction

Mammalian fertilization involves a complex series of activation events that start soon after the fusion of sperm with the egg (MII arrested oocyte) and lead to early embryonic development [1]. During this process, an increase in intracellular calcium (Ca^2+^) levels, driven by inositol 1,4,5-trisphosphate (IP_3_), plays the essential role in egg activation, preventing polyspermy and causing the resumption of meiosis [2,3]. In mammals, the intracellular Ca^2+^ signals in the ooplasm are characterized by long-lasting repetitive Ca^2+^ transients that begin minutes after sperm fusion and persist until pronuclear formation [2,4,5,6]. It is widely accepted that this increase in Ca^2+^ is mediated by the sperm-specific Phospholipase C zeta (PLCζ) [7]. Accumulating evidence, including microinjection of human recombinant protein and cRNA into human and mouse eggs, shows that PLCζ is sufficient to trigger Ca^2+^ oscillations similar to those observed at fertilization [7,8,9,10]. PLCζ catalyzes the hydrolysis of cytoplasmic stores of phosphatidylinositol 4,5-bisphosphate (PIP_2_) into IP_3_ and diacylglycerol (DAG). Elevation of IP_3_ levels trigger Ca^2+^ release from the endoplasmic reticulum [7,11]. PLCζ consists of two pairs of EF-hand domains at the N-terminus that play an essential role on its Ca^2+^ sensitivity [12,13,14], followed by the X and Y catalytic domains, essential for its enzymatic activity [7]. A charged and unstructured region, the XY-linker separates the X and Y catalytic domains [15]. Finally, there is a C2 domain at the C-terminus of PLCζ, which is essential for enzymatic activity and plays an important role in mediating membrane targeting via interactions with other phosphoinositides [16,17].

During the last two decades, several clinical and genetic reports have identified and linked PLCζ deficiencies, including reduced amounts or abnormal (mutant) forms, in patients with egg activation failure after Intracytoplasmic Sperm Injection (ICSI). Hence, these are specific cases of male factor infertility associated with egg activation [18,19,20,21,22,23]. Yoon et al. was the first to link fertilization failure with sperm expressing aberrant levels and disrupted localization patterns of PLCζ [18]. A year later, Heytens et al. reported the first PLCζ mutation located in the Y catalytic domain [19]. Injection of cRNA corresponding to this mutation in mouse eggs failed to induce Ca^2+^ oscillations leading to egg activation failure [19]. Since then, several other studies have identified over 25 PLCζ mutations located in different PLCζ domains leading to total fertilization failure (TFF) after ICSI [20,21,22,23,24]. TFF caused by reduced Ca^2+^ oscillations occurs in up to 1–3% of ICSI cycles [25,26]. Beyond TFF, PLCζ mutations have also been associated with polyspermy and early embryonic arrest [21,27,28]. PLCζ knockout mice were unable to trigger Ca^2+^ oscillations in mouse eggs and exhibited polyspermy [29,30]. These findings indicate the importance of PLCζ in ensuring monospermic fertilization. Moreover, reduced or absent PLCζ expression, combined with abnormal localization patterns observed at the sperm head, was strongly correlated with TFF [31].

**Figure 1:**
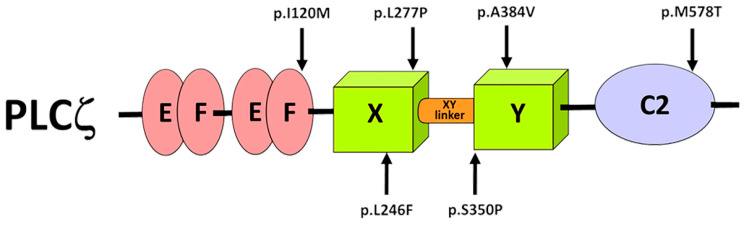
Schematic diagram of PLCζ structure including the location of mutations.

In the present study, we have introduced six human ICSI based infertility-linked PLCζ mutations (I120M, L246F, L277P, S350P, A384V and M578T) into the equivalent residues of human PLCζ sequence (Figure 1). These mutations were carefully selected because they span different functional domains of PLCζ, with the aim of providing a broader understanding of how alterations in distinct regions can affect its enzymatic activity and the resulting Ca^2+^ oscillations in oocytes. We combined in vivo functional characterization and in silico analysis to investigate the impact of these mutations, on the in vivo Ca^2+^ oscillation-inducing activity and structural stability of PLCζ. Despite the fact that previous experimental evidence suggested that PLCζ mutations lead to reduced or diminished enzymatic and Ca^2+^ oscillation-inducing activity, herein, we report for the first time a gain-of-function mutation within the catalytic domain of PLCζ, which results in egg activation failure. This suggests that the enzymatic activity of PLCζ may have to be within an optimal range, in order to ensure successful egg activation and thus early embryonic development. Finally, our collective findings confirm the indispensable role of all PLCζ domains in enzymatic activity and the successful induction of an appropriate pattern of Ca^2+^ oscillations required for egg activation.

## 2. Results

### 2.1. Monitoring of Ca^2+^ Oscillations of PLCζ Mutations in Mouse Eggs

**Figure 2:**
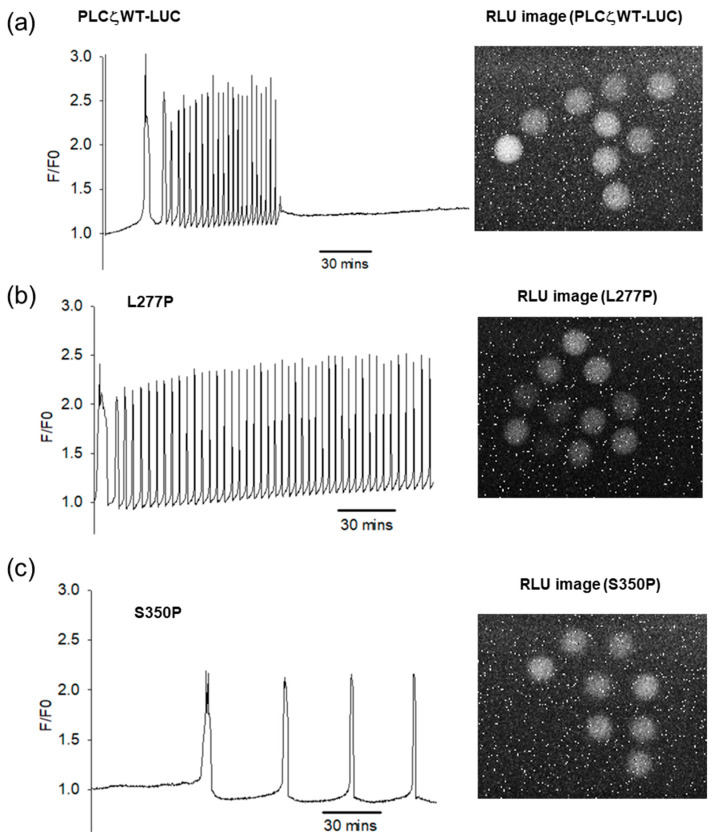
Fluorescence and luminescence measurements from mouse eggs after injection of PLCζ mRNA. Each trace shows Ca^2+^ oscillations record for the first 4 h after injection of mRNA. The fluorescence intensities are normalized to the staring values and hence plotted as the fluorescence (F) divided by the initial fluorescence (F0). Sample traces are shown for (**a**) the wild-type PLCζ; (**b**) PLCζ with the L277P mutation and (**c**) PLCζ with the S350P mutation. The images on the right-hand side show the luminescence of luciferase expression (10 min of integration) from the group of eggs from which the one OGBD trace on the left-hand side is plotted.

To determine the influence of PLCζ mutations on Ca^2+^ oscillations, cRNA corresponding to luciferase-tagged versions of human PLCζ^WT^ and the aforementioned mutants were microinjected into mouse eggs and the Ca^2+^ oscillations were monitored as previously described [10,13]. The relative luminescence units (RLUs) of every egg after injection were recorded at the end of 5 h to ensure comparable intracellular protein expression levels across all groups. Following a series of optimization experiments, our data revealed that microinjection of PLCζ^WT^ was able to induce high frequency and persistent Ca^2+^ oscillations (10 oscillations in the 1st hour), (Table 1 and Figure 2a). Almost all mutations showed a significant reduction in or complete abolishment of Ca^2+^ oscillations: I120M (6 oscillations in the 1st hour), L246F (0 oscillations in the 1st hour), S350P (2 oscillations in the 1st hour), A384V (1 oscillation in the 1st hour) and M578T (4 oscillations in the 1st hour) (Table 1 and Figure 2c). Interestingly, microinjection of PLCζ^L277P^ mutant, showed a significant increase in the Ca^2+^ oscillation-inducing activity of PLCζ (14 oscillations in the 1st hour); (Table 1 and Figure 2b). The Ca^2+^ oscillations with this mutation also started earlier and persisted for longer than control PLCζ^WT^ (Figure 2a,b), suggesting that the PLCζ^L277P^ mutant is considerably more efficacious than the PLCζ^WT^.

### 2.2. PLCζ Modeling and Predicting Impact of Mutation on PLCζ Stability

**Figure 3:**
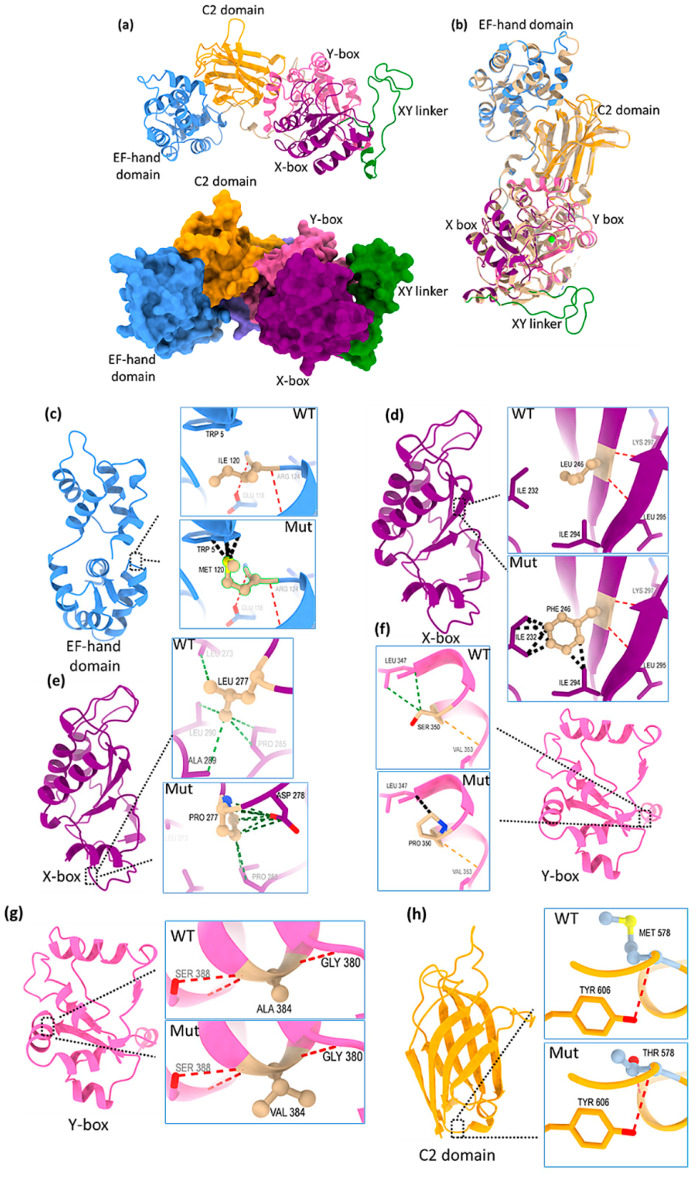
Overall structure of modeled PLCζ and the mutational analysis of PLCζ I120M, L246F, L277P, S350P, A384V, and M578T. (**a**) Cartoon and surface representation of the overall modeled full-length PLCζ structure. Protein is colored domain wise; EF-hand as blue, catalytic domain as purple (X-box) and pink (Y-box).The disordered XY linker region is shown as green. C2 domain is depicted in orange; (**b**) Structural superimposition of modeled PLCζ with chicken PLCζ in complex with calcium and phosphorylated threonine (PDBID: 9BCZ). The two structures are highly similar in overall fold; (**c**) I120M in EF-hand domain (in blue). The residues at 120 are shown in tan; (**d**) L246F in X-box of catalytic domain (in purple). The residues at 246 (WT and Mut) are shown in tan; (**e**) L277P in X-box of catalytic domain (in purple). The residues at 277 (WT and Mut) are shown in tan; (**f**) S350P in Y-box of catalytic domain (in pink). The residues at 350 (WT and Mut) are shown in tan; (**g**) Y-box of catalytic domain (in pink) contain A384V mutation. The residues at 384 (WT and Mut) are shown in tan; (**h**) M578T positioned in C2 domain (in orange). Contacts are shown as green dotted lines. Clashes are shown as black dotted lines. Hydrogen based interactions are shown as red dotted lines.

We have generated the predictive model of PLCζ using Swiss-Model [32]. We selected the chicken PLCζ as a template based on the highest global model quality estimation (GMQE) score of 0.85; quality estimation which combines properties from the target-template alignment. Alignment of chicken (*Gallus gallus*) and human (*Homo sapiens*) PLCζ sequences reveal 71.5% identity and 59.2% similarity (Supplementary Materials, Figure S2). The modeled structure contains three discrete domains, N-terminal EF-hand domain, followed by catalytic domain and C2 domain at the C-terminal. The EF-hand domain adopts helix-loop-helix topology (Figure 3a). The central catalytic domain has TIM-barrel-like topology and is divided into two boxes, X- and Y-box, connected through the XY-linker region (Figure 3a). The C-terminal C2 domain consists of antiparallel β-sandwich and is supposed to coordinate multiple Ca^2+^ ions. Structural comparison between modeled PLCζ and crystal structure of chicken PLCζ in complex with Ca^2+^ and phosphorylated threonine (PDBID: 9BCZ) showed that the structures are very similar, with an r.m.s.d. value of 0.147 Å (Figure 3b).

Mapping the mutation positions on the structure highlights clustering of these mutations in catalytic domain (X-box, Y-box and XY-linker). In addition, one mutation is located within the EF-hands and another one within the C2 domain. To evaluate the protein stability upon mutation, three structure-based predictors were used to calculate the difference in free energy of the mutation: delta delta G (ΔΔG). These tools revealed that while most of the mutations reduced the protein stability, two PLCζ mutations, the L277P and A384V showed significant stabilizing effects (Table 2).

### 2.3. Structural Analysis of PLCζ Mutants

The effect of intramolecular interactions upon mutation was analyzed by computing H-bonding, contacts and clashes in the wild-type and mutant PLCζ structures. UCSF ChimeraX was used to generate the mutated models of PLCζ for the corresponding amino acid substitutions. The I120M mutation is located at the C-terminal end of the EF-hand domain of PLCζ. In wild-type PLCζ, isoleucine at position 120 interacts with glutamate 118 and arginine 124 through main chain hydrogen bond. In the mutated protein (I120M), the main chain hydrogen bond remains the same with the neighboring residues (Figure 3c). However, in the mutated protein, due to methionine, there are several clashes to tryptophan 5 (5 clashes in mutated vs. 0 in wild-type) highlighting the destabilizing effect of methionine at position 120 on the protein structure.

Similarly, the substitution of leucine to phenylalanine at position 246 (L246F) in X-box of catalytic domain creates steric hindrance due to the bulkier side of phenylalanine. Although the mutation is conservative with a non-polar residue replacing another non-polar residue, the interaction profile changes due to slightly different side chain. There are 7 clashes to neighboring isoleucine 232 and isoleucine 294 (Figure 3d).

In PLCζ catalytic domain X-box region, at position 277, substitution of leucine to proline would lead to gain of crucial interaction with aspartate 278 (Figure 3e). Leucine as a hydrophobic residue interacts with neighboring hydrophobic residues like leucine 273, alanine 289 and leucine 290. Proline pyrrolidine ring participates in carbonyl interaction with adjacent residues aspartate 278 and stabilizes the structure consolidating the stabilizing effect found by structure-based stability prediction tools (Table 2).

In the Y-box of catalytic domain, substitution of the serine at position 350 to proline (S350P) leads to clashes with the neighboring leucine 347 residue. Although the hydrogen bonding in wild-type and mutated residue remain the same, serine participates in hydrophobic interaction with leucine 347, while proline clashes with leucine 347 (Figure 3f).

At position 384, alanine to valine substitution (A384V), (both the residues are hydrophobic, non-polar) seems not to affect the hydrogen-bonding network (Figure 3g). Valine may provide additional contacts compared to alanine (14 contacts in wild-type alanine vs. 29 contacts in valine). This may explain the stabilizing outcome of structure-based predictors.

The M578T mutation is located within the C2 domain. This substitution, which involves similar kinds of residues, may not hamper their hydrogen bonding and both methionine and threonine make hydrogen bond with tyrosine 606 (Figure 3h). In the case of mutant, threonine makes 10 contacts compared to 12 contacts of the wild-type methionine.

## 3. Discussion

In this study, we characterized six infertility-linked PLCζ mutations previously identified in patients with oocyte activation failure. The six mutations are located in different PLCζ domains. The in vivo Ca^2+^ oscillations-inducing activity of PLCζ mutants was tested by microinjection of cRNA into mouse eggs, by monitoring Ca^2+^ transients, as previously described [10]. To quantify and ensure comparable expression levels for PLCζ wild-type and mutants, a firefly luciferase (LUC) reporter was expressed at the C-terminus of PLCζ constructs. Microinjection of wild-type PLCζ resulted in high-amplitude of Ca^2+^ transients that started 30 min post injection and persisted for 2.5 h (Figure 2a). The amplitude and duration of these oscillations were consistent with previous reports and resembled Ca^2+^ transients observed during fertilization [7,33]. To achieve optimal and comparable expression levels, varying amounts of cRNA were injected (Table 1). Our experiments revealed that all PLCζ mutations, except L277P, led to abnormal Ca^2+^ transients, ranging from significantly reduced and delayed oscillations to a complete loss of activity (Figure 2b,c, Supplementary Materials Figure S1 and Table 1).

To assess the structural impact of PLCζ mutations, a Swiss-Model of human PLCζ was generated using chicken PLCζ as a template. Structural comparison between the modeled human PLCζ and the chicken PLCζ crystal structure revealed high similarity. Structural modeling predicted that all mutations, except L277P and A384V, destabilize the protein structure (Table 2). Moreover, analysis of generated intramolecular interactions highlighted the destabilizing effect of these mutants on enzyme activity (Figure 3c,d,f,h).

Torra-Massana et al. identified the novel heterozygous I120M mutation, located between the EF-hand domain and X-catalytic domain (C-terminal end of EF-hand), in a couple who experienced ICSI failure due to TFF [21]. Functional assays revealed that this mutation could induce egg activation, with PN observed in 90% of the injected eggs. Furthermore, in silico analysis predicted that I120M had no destabilizing effect on PLCζ structure. In contrast, our findings showed a significant reduction in Ca^2+^ oscillations upon cRNA injection (Table 1). Additionally, our in silico studies revealed severely reduced protein stability (Table 2 and Figure 3c). These discrepancies may be due to differences in experimental conditions, microinjection protocols or the specific endpoints assessed, as measuring overall activation rates may not fully reflect subtle alterations in oscillation dynamics. Together, our combined functional and computational data offer additional mechanistic insight into how the I120M mutation may modulate PLCζ activity, highlighting the importance of evaluating both egg activation outcomes and detailed oscillation profiles. It is also worth noting that this mutation was discovered in a normozoospermic patient, who failed ICSI repeatedly and was able to achieve fertilization only using assisted egg activation—ICSI.

The EF-hand domain located at the N-terminus of PLCζ plays an essential regulatory role in its enzymatic activity. Swapping the EF-hand domain of mouse and rat confirmed the important regulatory role of this domain in PLCζ activity [34]. Kuroda et al. demonstrated that deleting of any of the four EF-hand domains have abolished the nuclear translocation ability of PLCζ, thereby disrupting its ability of PLCζ to induce Ca^2+^ oscillations in a cell-cycle dependent manner [35]. It is highly likely that mutations within the EF-hand region might impair Ca^2+^ oscillations by either prolonging or shortening the onset of oscillations.

Mutations in the catalytic domains of PLCζ have been associated with TFF after ICSI [21,22,36]. In this study, we characterized four missense mutations located in the X and Y catalytic domains (L246F, L277P, S350P, and A384V) identified in patients with recurring ICSI failures. Dai et al. identified L246F and S350P homozygous mutations [22]. Sperm of both mutants exhibited abnormal PLCζ localization patterns. In silico analysis revealed that both mutations altered hydrogen bonds of residues 246 and 350, destabilizing the protein structure. Our results showed that both mutants resulted in severe reduction or complete abolishment of Ca^2+^ oscillations (Table 1, Figure 2c). Moreover, in silico analysis revealed that both mutants destabilized PLCζ structure (Table 2). At position 246, substitution of leucine with phenylalanine caused steric hindrance (Figure 3d), while at position 350, substitution of serine with proline induced clashes, reducing structural stability (Figure 3f).

The A384V homozygous mutation, located in the Y-catalytic domain of PLCζ, was identified in two patients with polyspermy [23,28]. Peng et al. revealed that PLCζ mutations in the catalytic domain are linked to fertilization failure and polyspermy [27]. Moreover, PLCζ knockout (KO) mouse models of PLCζ exhibited polyspermy following IVF [29,30]. Our in silico analysis revealed that A384V stabilizes PLCζ structure (Table 2 and Figure 3g). However, microinjection of A384V cRNA completely abolished Ca^2+^ oscillations within the first hour of injection (Table 1). The mutant inability to induce Ca^2+^ oscillations may explain its association with polyspermy, since Ca^2+^ oscillations are essential for initiating cortical granule exocytosis; an important step that ensures monospermic fertilization [3].

The PLCζ L277P homozygous mutant, located in the X catalytic domain was first identified in a patient who failed ICSI due to TFF [23]. Molecular modeling suggested that this mutation disrupts protein folding and weakens enzymatic activity. Moreover, injection of L277P cRNA into human eggs resulted in egg activation deficiency. Our findings demonstrate for the first time that a PLCζ mutation can stabilize PLCζ structure. Microinjection of L277P cRNA into mouse eggs triggered significantly more Ca^2+^ oscillations, which persisted for over 4 h compared to wild-type PLCζ (Figure 2b and Table 1). Similarly, in silico predictions revealed that the mutation enhanced the stability via forming a crucial interaction with aspartate 278 (Table 2 and Figure 3e). The amplitude and duration of PLCζ-induced Ca^2+^ oscillations are essential for successful fertilization and early embryonic development [37,38]. The lack of sufficient Ca^2+^ oscillations is associated with poor embryonic development, reduced inner cell mass, as well as increased expression of apoptotic genes [39]. Excessive stimulation of Ca^2+^ release, on the other hand, is equally deleterious in affecting gene expression and hindering blastocyst development [40]. It is likely that the prolonged Ca^2+^ transients observed with L277P may explain its association with early embryonic arrest. Notable, we have previously shown that injection of levels PLCζ^WT^ into mouse eggs that cause high frequency Ca^2+^ oscillations is associated with a failure of embryos to reach the blastocyst stage [38].

Our findings demonstrated that the M578T mutation, located at the C2 domain of PLCζ, has significantly reduced Ca^2+^ oscillations by 60% (Table 1). Several studies have reported that mutations affecting this domain are associated with TFF [21,28]. Our in silico analysis predicted that this mutation severely destabilizes PLCζ structure. Our findings align with two prior studies that identified the M578T mutation and proposed that it disrupts a hydrogen bond to K580, thereby hindering the interaction of nearby hydrophobic residues leading to a defective PLCζ [23,24]. Moreover, the catalytic activity of M578T mutation was significantly reduced when compared to wild-type PLCζ [24]. Yuan et al. measured PLCζ activity using the synthetic substrate *p*-nitrophenylphosphorylcholine (NPPC), which is hydrolyzed into *p*-nitrophenol [24]. However, a key limitation of this assay is that it measures phosphorylcholine rather than PIP_2_ hydrolysis, the physiological substrate of PLCζ. Previously we were able to measure the PLCζ hydrolytic activity using radiolabeled PIP_2_ [13,41]. However, this method is no longer available due to technical limitations and discontinuation of the kit. Therefore, a commercial colorimetric kit measuring the hydrolysis of PIP_2_ would be essential as a reliable tool to evaluate PLCζ activity. If developed, such a kit might be used as a diagnostic marker for cases of unexplained male infertility linked to defective PLCζ.

It is important to note that the sensitivity of mouse eggs is approximately 10-fold higher to IP_3_-induced Ca^2+^ release when compared to human eggs [42,43]. This might be due to the fact that ATP concentration is almost two-fold higher in mouse eggs than in human eggs [43]. The higher ATP concentration observed in mouse eggs compared to human eggs might explain the higher sensitivity of mouse eggs to PLCζ microinjection in comparison to human eggs [44]. This implies that the observed low frequency Ca^2+^ oscillations of PLCζ mutants in mouse eggs might fail to trigger any Ca^2+^ oscillations in human eggs. Therefore, extrapolating these results to human fertilization should take into account species-specific differences in egg sensitivity.

Moreover, while our in silico analyses provide valuable insights into how each mutation might affect PLCζ structure and stability; we acknowledge that such computational predictions cannot substitute for direct structural validation. In this study, we integrated these modeling tools, using the chicken PLCζ crystal structure as a novel template to complement our functional data and offer a plausible mechanistic interpretation of the observed effects. Nonetheless, these findings should be considered indicative rather than definitive and future studies employing experimental structural approaches such as X-ray crystallography or cryo-EM of human PLCζ protein will be essential to confirm and extend these predictions.

In summary, our functional and structural analysis demonstrates that PLCζ mutations across all domains disrupt Ca^2+^ signaling, leading to failure of fertilization. Our findings support the use of PLCζ as a diagnostic marker for investigating cases of male unexplained infertility. For the first time, our study reported a stabilizing PLCζ mutation that causes hyperstimulation of Ca^2+^ transients. This finding further confirms that successful fertilization requires fine-tuning, since low or high Ca^2+^ transients are equally deleterious to fertilization. For the first time, we used the crystal structure of chicken PLCζ as a template for molecular modeling, which could serve as a superior prediction tool for structural analysis. In all investigated mutations, assisted egg activation-ICSI using Ca^2+^ ionophores like A23187 were used to help overcome TFF. The recombinant PLCζ protein might represent a superior therapeutic intervention for treating cases of egg activation failure. Recently, we were able to develop a recombinant PLCζ protein that was enzymatically active and capable of inducing Ca^2+^ oscillations similar to those observed at fertilization [41].

## 4. Materials and Methods

### 4.1. Plasmid Construction and cRNA Synthesis

PLCζ mutants were generated by site-directed mutagenesis [GenScript Biotech (Piscataway, NJ, USA)] and were cloned into pCR3-LUC plasmid vector [10]. All PLCζ mutants were amplified by polymerase chain reaction (PCR) using Phusion polymerase (Thermofisher, Waltham, MA, USA) and the appropriate primers to incorporate a 5′-BamHI site and a 3′-NotI site and were cloned into a modified pCR3-LUC. The primers used for the amplification of PLCζ mutants were 5′-ACCCGGATCCATGGAAATGAGATGGTTTTTGTC-3′ (forward) and 5′-CCAAGCGGCCGCACATCTGACGTACCAAACATAAAC-3′ (reverse). All the plasmids were linearized and cRNA synthesis was performed as previously described [45] using the mMESSAGE mMACHINE T7 transcription Kit (Invitrogen, Waltham, MA, USA) and a poly(A)tailing Kit (Invitrogen, Waltham, MA, USA) as per manufacturer’s instructions.

### 4.2. Microinjection of PLCζ mRNA in Mouse Eggs

Mature metaphase II oocytes (referred to as eggs) were collected from super-ovulated CD-1 female mice as describe elsewhere [46]. All animal experiments complied with ARRIVE guidelines and were carried out in accordance with the U.K. Animals (Scientific Procedures) Act 1986, EU Directive 2010/63/EU, and the National Institutes of Health Guide for the Care and Use of Laboratory Animals (NIH Publications No. 8023, revised 1978). Eggs were microinjected as described previously [47] using KCl-HEPES buffer (100 mM KCl, 20 mM HEPES, pH 7.4) containing the mRNA which was then mixed 1:1 with a 1mM of Oregon Green BAPTA dextran (OGBD) before injection. Eggs were maintained during imaging in drops of HKSOM medium, with a concentration of D-luciferin of 1mM as described previously [38]. The drops of medium were covered with mineral oil. The dish with eggs was heated and held on the stage of a Zeiss Axiovert 100 microscope inside a dark box as described previously [46]. Images were collected using a Retiga-LUMO CCD camera. OGBD fluorescence (Ca^2+^ oscillations) were recorded for 5 h and then the luminescence was recorded for 30 min (in 10 min time integration windows) to assay the luciferase expression in relative light units (RLU). Image data was analyzed using ImageJ version 1.54p (Wayne Rasband, National Institutes of Health, Bethesda, MD, USA) and SigmaPlot version 12 (Systat Software, Inc., San Jose, CA, USA). Fluorescence and luminescence background area the same sizes as eggs were subtracted from images in order to quantify signals. For statistical analysis, an unpaired Student’s *t*-tests was used, in order to compare each mutant individually against the wild-type control, as our primary aim was to assess the effect of each mutation relative to normal PLCζ function rather than to compare mutants directly with each other. All statistical analyses were performed using SigmaPlot version 12.

### 4.3. Modeling of PLCζ and Structural Impact Assessment of Infertility-Linked Mutations

In absence of human PLCζ structure in protein data bank (PDB), we generated the structure model by Swiss-Model [32]. Template search matching of human PLCζ protein sequence was performed against the Swiss-Model template library. Based on the protein sequence coverage and sequence identity, chicken PLCζ in complex with Ca^2+^ and phosphorylated threonine (PDBID: 9BCZ) was selected for homology modeling. The predicted model’s quality was assessed using MolProbity tool [48], which evaluated phi, psi, and Cβ deviations and generated a Ramachandran plot to map the backbone conformational space. Quaternary structure analysis was performed with QSQE, a tool that predicts the accuracy of the quaternary arrangement by integrating interface conservation scores, structural clustering, and interface descriptors [49].

To investigate the PLCζ stability, we used three structure-based computational tools for a predictive in silico analysis of the impact of the mutations on the structure and function of PLCζ. These included a mutation cut off scanning matrix (mCSM) [50], Site-Directed Mutator (SDM) [51] and CUPSAT [52]. These are efficient and versatile tools to predict changes in protein stability upon point mutations. To understand the intramolecular interactions upon mutations, we have generated the mutant PLCζ. The mutations were introduced into the modeled PLCζ structure and the interactions were analyzed across the wild and mutant protein molecules using the structural analysis software program UCSF ChimeraX [53].

## Figures and Tables

**Table 1:** Summary of Ca^2+^ oscillation-inducing activity in the first hour of different PLCζ mutants in comparison to wild-type PLCζ.

Mutation	Injection Concentration	RLU	Number of Ca^2+^ Oscillations (First Hour After First Peak)	Mean Value	Std. Dev. (Number of Ca^2+^ Oscillations)	*p* Value (Compare to PLCζ^WT^-LUC)
PLCζ^WT^-LUC	189 ng/µL	81 (9 eggs)	8–11	9.63	1.40	-
I120M	31.5 ng/µL	79 (6 eggs)	4–6	5.83	0.98	*p* < 0.001
L246F	16.5 ng/µL	55 (10 eggs)	0	0	0	*p* < 0.001
L277P	60.5 ng/µL	76 (11 eggs)	10–20	13.22	3.11	*p* < 0.01
S350P	4.5 ng/µL	80 (8 eggs)	2	2.00	0	*p* < 0.001
A384V	29.6 ng/µL	66 (9 eggs)	0–2	1.00	0.76	*p* < 0.001
M578T	155.0 ng/µL	101 (5 eggs)	3–5	4.00	0.82	*p* < 0.001

**Table 2:** Predicted protein stability scores of the mutants by the three structure-based predictors. ΔΔG values of energies are given in kcal/mol. If the mutation destabilizes the structure, ΔΔG is increased, whereas stabilizing mutations decrease the ΔΔG.

S. No	Mutations	Predicted ΔΔG (kcal/mol)			Outcome/Overall Stability
		mCSM	SDM	CUPSAT	
1	WT	00	00	00	--
2	I120M	−1.33	−1.26	−0.02	Destabilizing
3	L246F	−1.68	−0.66	−2.19	Destabilizing
4	L277P	−0.76	0.08	0.03	Stabilizing
5	S350P	−0.49	−0.98	−1.77	Destabilizing
6	A384V	0.33	−1.03	1.24	Stabilizing
7	M578T	−2.41	−2.18	−1.10	Destabilizing

## Data Availability

Data is contained within the article.

## Supplementary Materials

The following supporting information can be downloaded at https://www.mdpi.com/article/10.3390/ijms26136241/s1.

## Abbreviations

The following abbreviations are used in this manuscript:

TFF	Total Fertilization Failure
ICSI	Intracytoplasmic Sperm Injection
PIP_2_	Phosphatidylinositol 4,5-Bisphosphate
IP_3_	Inositol Trisphosphate
Ca^2+^	Calcium ions
DAG	Diacylglycerol
PLCζ	Phospholipase C zeta
