# Supplementary material for: A Novel PLCζ Mutation Linked to Male Factor Infertility Induces a Gain-of-Function Effect on Ca2+ Oscillations in Eggs
Source: Int J Mol Sci. 2025 Jun 28;26(13):6241. doi: 10.3390/ijms26136241 (PMC12249980; doi:10.3390/ijms26136241)
Supplement: Supplementary file 1 [file ijms-26-06241-s001.zip › ijms-3663295-supplementary.pdf]

SUPPLEMENTARY MATERIALS

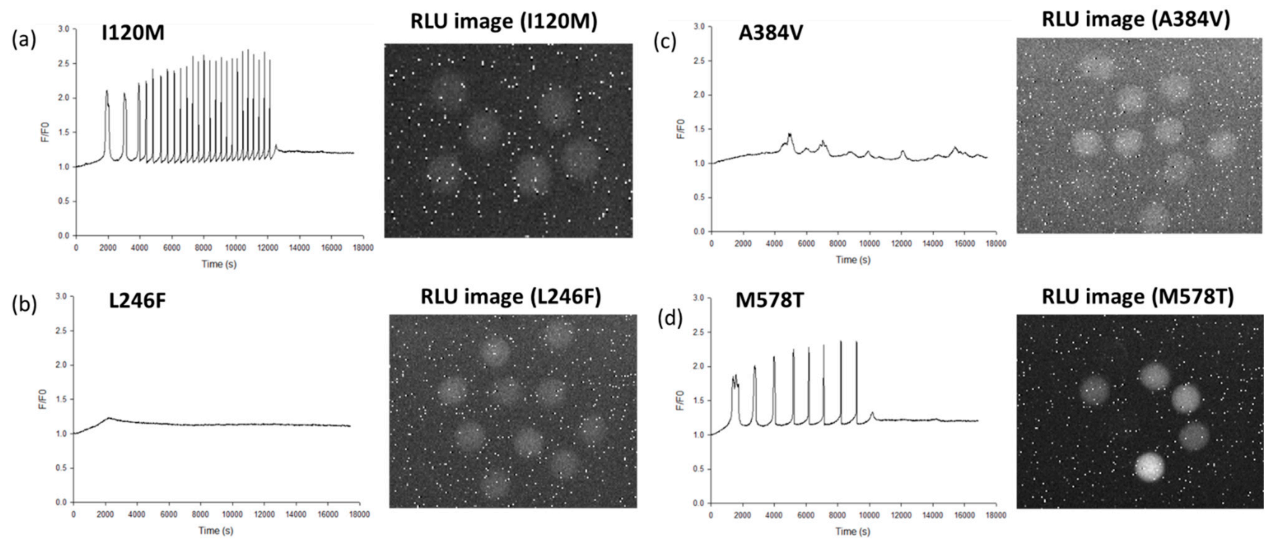

Figure S1. Fluorescence and luminescence measurements from mouse eggs after injection of PLCζ mRNA. Each trace shows Ca^{2+} oscillations record for the first 4 hours after injection of mRNA. The fluorescence intensities are normalized to the staring values and are therefore plotted as the fluorescence (F) divided by the initial fluorescence (F0). The sample traces are shown for (a) PLCζ with the I120M mutation; (b) PLCζ with the L246F mutation; (c) PLCζ with the A384V mutation and (d) PLCζ with the M578T mutation. The images on the right-hand side of each panel show the luminescence of luciferase expression.

```
Gallus gallus      1 MEENRWFLNIIQDGFMNGKIDFDSTVKLLEKLHMPFNLAHVKHVFKKTVD      50
                      .|.||||:.|||.|..|||:.:.|.:|||||.:..:..|||.:||.. |
Homo sapiens       1 -MEMRWFLSKIQDDFRGGKINLEKTQRLLEKLDIRCSYIHVKQIFKDN-D      48

Gallus gallus     51 KRKIHTINIEDFRAIYRAIVHRNEFHEIFCAYSENRKNLADTELTAFLKK     100
                     :.|...|.||:||||||.|.||.|..|||..||||||.|..:.|..||.:
Homo sapiens      49 RLKQGRITIEEFRAIYRIITHREEIIEIFNTYSENRKILLASNLAQFLTQ      98

Gallus gallus    101 EQFKTEGAETTALEVILKYEPIDEVRKRRQLSFEGFIRYMSSEDCTIFKK     150
                     ||:..|.::..|.|:|.|||||:||||..|:|.|||.|||.|.:|.:||.
Homo sapiens      99 EQYAAEMSKAIAFEIIQKYEPIEEVRKAHQMSLEGFTRYMDSRECLLFKN     148

Gallus gallus    151 EHRTVYQDMNHPLCDYFISSSHNTYLVSDQLIGPSDLNGYISALLKGCRC     200
                     |.|.|||||.|||.|||||||||||||||||:|||||.||:|||:|||||
Homo sapiens     149 ECRKVYQDMTHPLNDYFISSSHNTYLVSDQLLGPSDLWGYVSALVKGCRC     198

Gallus gallus    201 LEIDCWDGSNNDPVVYHGHTLTSKITFCSVIHVVDKYAFAASDYPVVLSL     250
                     ||||||||:.|:||||||:|||||:.|.:||..:.||||..|||||||||
Homo sapiens     199 LEIDCWDGAQNEPVVYHGYTLTSKLLFKTVIQAIHKYAFMTSDYPVVLSL     248
```

```
Gallus gallus    251 ENHCSTKQQERIAQYLLNILGDKLLTSPIGDIEVTQLPSPEALKFKILVK    300
                     ||||||.|||.:|..|....|:.||:..:.|...| ||||||||||||||
Homo sapiens     249 ENHCSTAQQEVMADNLQATFGESLLSDMLDDFPDT-LPSPEALKFKILVK    297

Gallus gallus    301 NKKCGTIEETMLRKGRDSHGETGEVSEEEITSSDEETD-EKTP---LYPK    346
                     |||.||::||..|||.|..|:          :.|:||. :|.|   |:
Homo sapiens     298 NKKIGTLKETHERKGSDKRGD----------NQDKETGVKKLPGVMLF--    335

Gallus gallus    347 SGSSKRKSEGRSSPPPRKKAKVKKMKIAMGLSDLVIYTKSEKFVSFEHSL    396
                                      ||.|.:|:|||:.|||||||||:|||.||:||.
Homo sapiens     336 -----------------KKKKTRKLKIALALSDLVIYTKAEKFKSFQHSR    368

Gallus gallus    397 AHQKCYENNSIGELKAQKFVKHAANQFVSHTSRFITRIYPKGTRAGSSNY    446
                     .:|:..|||||||.:|:|..|...::|:.||.:||||||||.|||.|||:
Homo sapiens     369 LYQQFNENNSIGETQARKLSKLRVHEFIFHTRKFITRIYPKATRADSSNF    418

Gallus gallus    447 NPQEFWNVGCQMVALNFQTSGTPMELQNGKFLDNGGCGYILKPEFLRNRN    496
                     |||||||:|||||||||||.|.||:|||||||||||.||||||.|||...
Homo sapiens     419 NPQEFWNIGCQMVALNFQTPGLPMDLQNGKFLDNGGSGYILKPHFLRESK    468

Gallus gallus    497 STFNPHNVGRYSNPLSLSIRLISGHQLPPSNLSKSNKADPLVQLEIYGVP    546
                     |.|||.|: :...|::|:||||||.|||.:: |.|||.|.||.:|::|||
Homo sapiens     469 SYFNPSNI-KEGMPITLTIRLISGIQLPLTH-SSSNKGDSLVIIEVFGVP    516

Gallus gallus    547 EDQAKRKSSVIKSNALSPRWDETFSFTVQVPELALIRFCVQDEISLVAND    596
                     .||.|:::.|||.||.||||:|||:|.:.:.|||||||||.|:.:..:..|:
Homo sapiens     517 NDQMKQQTRVIKKNAFSPRWNETFTFIIHVPELALIRFVVEGQGLIAGNE    566

Gallus gallus    597 FLGQYTLPLLSLSKGYCTVPLFSKSGGKLEPASLFVYVWYY-            637
                     ||||||||||.::|||..:||||:.|..||||||||||||.
Homo sapiens     567 FLGQYTLPLLCMNKGYRRIPLFSRMGESLEPASLFVYVWYVR            608
```

Figure S2. Alignment of chicken (*Gallus gallus*) and human (*Homo sapiens*) PLCζ sequences reveal 71.5% identity and 59.2% similarity.
